# Supplementary figures and images for: MicroRNA, mRNA, and Proteomics Biomarkers and Therapeutic Targets for Improving Lung Cancer Treatment Outcomes
Source: Cancers (Basel). 2023 Apr 14;15(8):2294. doi: 10.3390/cancers15082294 (PMC10137184; doi:10.3390/cancers15082294)

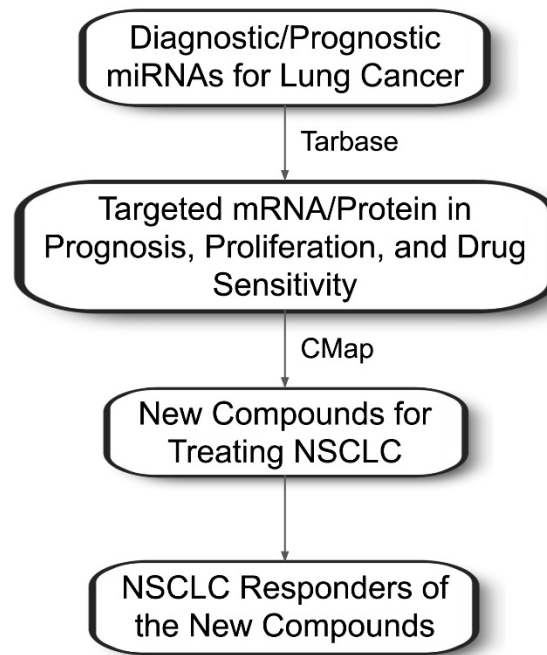

**Figure S1.** Overall study scheme. The arrows indicate analysis flow.

Supplement: Supplementary file 1 [file cancers-15-02294-s001.zip › S1. Overall flowchart.pdf]
